# 

**Published:** 1888-10

**Authors:** 


### CONTENTS.

COMMUNICATIONS.

Anæsthetics in Dental Practice........................................ 473
Short Notes on the Examination of the Teeth for Bacteria, etc........ 477

PROCEEDINGS.

Georgia State Dental Society........................................ 482
National Association of Dental Faculties........................................ 497
National Association of Examiners........................................ 502

SELECTIONS.

Reflex Pain following the Extraction of a Tooth........................................ 505
Dental Caries and Reflex Paralysis........................................ 506
The Treatment of Ulcers........................................ 508
What Cocaine to Use........................................ 508
How to tell a Horse's Age........................................ 509
A Centenarian........................................ 511
Early Rising and Longevity........................................ 511
Who is my Neighbor?........................................ 513
Bright's Disease........................................ 514
The Spread of Antisepsis........................................ 514

SOCIETY NOTES.

Central Illinois Dental Society........................................ 516
Obituary........................................ 518
Salmagundi........................................ 520

